# Antibacterial and Antibiofilm Activities of Nonpolar Extracts of* Allium stipitatum* Regel. against Multidrug Resistant Bacteria

**DOI:** 10.1155/2018/9845075

**Published:** 2018-07-11

**Authors:** Arunkumar Karunanidhi, Ehsanollah Ghaznavi-Rad, Rukman Awang Hamat, Mallikarjuna Rao Pichika, Leslie Than Thian Lung, Fazlin Mohd Fauzi, Sridevi Chigurupati, Alex van Belkum, Vasanthakumari Neela

**Affiliations:** ^1^Department of Medical Microbiology and Parasitology, Faculty of Medicine and Health Sciences, Universiti Putra Malaysia, 43400 Serdang, Selangor Darul Ehsan, Malaysia; ^2^Department of Pharmacology and Chemistry, Faculty of Pharmacy, Universiti Teknologi MARA, 42300 Bandar Puncak Alam, Selangor Darul Ehsan, Malaysia; ^3^Department of Microbiology and Immunology, Faculty of Medicine, Arak University of Medical Sciences, Basij Square, Arak 38481-7-6941, Iran; ^4^Department of Pharmaceutical Chemistry, School of Pharmacy, International Medical University, No. 126, Jalan Jalil Perkasa 19, Bukit Jalil, 57000 Kuala Lumpur, Malaysia; ^5^Department of Medicinal Chemistry and Pharmacognosy, College of Pharmacy, Qassim University, Buraidah 51452, Saudi Arabia; ^6^La Balme Microbiology Unit, BioMerieux, 3 route de Port Michaud, 38390 La Balme-les-Grottes, France

## Abstract

The present study assessed the* in vitro *antibacterial and antibiofilm potential of hexane (ASHE) and dichloromethane (ASDE) extracts of* Allium stipitatum *(Persian shallot) against planktonic cells and biofilm structures of clinically significant antibiotic resistant pathogens, with a special emphasis on methicillin-sensitive* Staphylococcus aureus *(MSSA), methicillin-resistant* S. aureus *(MRSA), and emerging pathogens,* Acinetobacter baumannii* and* Stenotrophomonas maltophilia*. Antibacterial activities were determined through disk diffusion, minimum inhibitory concentration (MIC), minimum bactericidal concentration (MBC), time-kill kinetics, and electron microscopy. Antibiofilm activity was assessed by XTT [2,3-*bis*(2-methoxy-4-nitro-5-sulfo-phenyl)-2H-tetrazolium-5-carboxanilide] reduction assay and by confocal laser scanning microscopy (CLSM). The zone of inhibition ranged from 13 to 33 mm, while the MICs and MBCs ranged from 16 to 1024* μ*g mL^−1^. Both ASHE and ASDE completely eradicated overnight cultures of the test microorganisms, including antibiotic resistant strains. Time-kill studies showed that the extracts were strongly bactericidal against planktonic cultures of* S. aureus*, MRSA,* Acinetobacter baumannii,* and* S. maltophilia* as early as 4 hours postinoculation (hpi). ASHE and ASDE were shown to inhibit preformed biofilms of the four biofilm phenotypes tested. Our results demonstrate the potential therapeutic application of ASHE and ASDE to inhibit the growth of gram-positive and gram-negative biofilms of clinical significance and warrant further investigation of the potential of* A. stipitatum *bulbs against biofilm-related drug resistance.

## 1. Introduction

Biofilms are ubiquitous in nature which can be found at the bottom of streams to extremely hot waters of hot springs. In human health, biofilms pose a great threat due to their resistant nature to antibiotics, contamination of indwelling medical devices, forming dental plaques causing tooth decay, chronicity in wounds, and association with several illnesses from cystic fibrosis to otitis media. Biofilm forms of microbes are up to 1000-fold more resistant to antibiotics than their planktonic counterparts [1, 2].

Infectious diseases caused by multidrug resistant (MDR) pathogens are the biggest challenge in healthcare. The conditions are made more worrisome by the biofilm associated infections. Such diseases are most frequently caused by* S. aureus*,* S*.* epidermidis*,* Pseudomonas aeruginosa*,* A. baumannii*, and Enterobacteria such as* Escherichia coli *[3–5].

Few novel antibacterial agents have been developed in recent years and their bacteriostatic or bactericidal activity results in selective pressure, with antimicrobial resistance as an unavoidable aftermath of their use [6]. In addition, effective therapies which target bacterial biofilms are scarce due to their implicit resistance to antibiotics [7]. An extensive review by Wu et al. [8] has shared the clinical experiences/challenges in managing biofilm infections. If the infections are not involving foreign body, a high dose of antibiotics or combinational therapy may sometimes eliminate the infections. But in cases where foreign bodies such as catheters, shunts, heart valves, pacemakers, stents, breast implants, intrauterine devices, contact lenses, etc. are used, the removal of material, followed by treatment is often necessary.

To date, several agents from natural products to synthetic compounds have been routinely used or evaluated for treating biofilm infections [9–13]; however, the integrity of the cells in the biofilm matrix and reduced permeability to antibiotics limit the complete detachment or destruction of biofilms. Hence, the search for new antimicrobials and biofilm dissolving agents is always vital in the human medicine.

*Allium stipitatum *that belongs to the Amaryllidaceae family is a wild edible plant mostly found in the cold mountains of central, south, and western Iran as well as some provinces of Turkey and central Asia [14, 15]. Persian shallot is locally known as “Mooser” in Iran and widely used as a spice and flavoring agent in Persian foods, especially as added ingredients to yogurt, salads, and pickling mixtures. The appearance, shape, color, weight, texture, and storage tissues differentiate* A. stipitatum *from the common shallot,* A. ascalonicum* [15, 16]. Unlike other* Allium* members, Persian shallot usually consists of a single bulb and rarely two bulbs [15]. Dried bulbs of* A. stipitatum* are frequently used in Persian folklore medicine for various ailments, like anti-inflammatory disorders, diarrhoea, gout, haemorrhoids, psoriasis, rheumatic arthritis, stomach pain, etc. [17]. Several studies performed on* A. stipitatum* elucidated its antibacterial, antiproliferative, anthelmintic, antiprotozoal, immunomodulatory, and wound healing properties [18–23]. Moreover, in our recent study, we have shown that* A. stipitatum *exerts anti-MRSA and wound healing activity in a mouse model of burn wound and infection [24]. We have also reported that both the extracts were relatively noncytotoxic towards mammalian cells and effective in eradicating MRSA colonized in thermal wounds. However, the effect of* A. stipitatum* on bacterial biofilm formation is still not clearly defined, requiring further investigation. In this study, we investigated the effects of ASHE and ASDE against a panel of medically important gram-positive and gram-negative bacteria followed by SEM and TEM examination of the* in vitro* effects of ASHE and ASDE on bacterial cells at different concentrations. We further provide evidence that ASHE and ASDE can increase the susceptibility of bacterial biofilms with emphasis on MSSA, MRSA,* A. baumannii*, and* S. maltophilia* biofilms.

## 2. Materials and Methods

### 2.1. Bacterial Strains and Culture Conditions

Test microorganisms* Acinetobacter baumannii* ATCC 19606,* Acinetobacter lwoffii*, Enterobacter spp.,* Escherichia coli* ATCC 25922,* Klebsiella pneumoniae*, methicillin-resistant* Staphylococcus aureus* (MRSA) ATCC 43300,* Staphylococcus aureus* ATCC 25923,* Pseudomonas aeruginosa* ATCC 27853,* Salmonella typhi*,* Shigella dysenteriae*,* Stenotrophomonas maltophilia* ATCC 13637, and vancomycin resistant enterococci (VRE) were obtained from the Medical Microbiology and Parasitology Laboratory at Universiti Putra Malaysia (UPM). All strains were confirmed by cultural and biochemical characteristics and maintained in glycerol stock cultures at -80°C prior to use. Bacterial cultures were propagated by streaking onto tryptic soy agar (TSA) or nutrient agar (NA). Single colonies of bacteria from the overnight cultures were inoculated into Luria-Bertani (LB) broth or brain heart infusion broth (BHI) and incubated in a shaking incubator at 37°C.

### 2.2. Chemicals

Merck supplied dimethyl sulfoxide (DMSO) and bacterial growth media [brain heart infusion (BHI) broth, Mueller-Hinton agar (MHA), tryptic soy broth (TSB), Mueller-Hinton broth (MHB), and Luria-Bertani (LB) broth]. Resazurin (7-hydroxy-3H-phenoxazin-3-one 10-oxide, monosodium salt) and phosphate-buffered saline (PBS) were purchased from Fisher Scientific (M) Sdn Bhd, Malaysia. Antibiotic discs and powder were purchased from Oxoid Limited, Hampshire, UK. Filter paper discs (6 mm diameter) were purchased from GE Healthcare, Malaysia; sterile swabs with wooden handle (FisherbrandTM) were purchased from Thermo Fisher Scientific Sdn. Bhd, Malaysia; and 96-well polystyrene microtitre plates (©TPP, Trasadingen, Switzerland) were obtained from NeoScience Sdn. Bhd, Malaysia. Resazurin was prepared as a stock solution of 100* μ*g mL^−1^ and was used at a final concentration of 0.01% (w/v) in PBS (pH 7.2). The stock solution was filter sterilized in a 0.20* μ*m pore filter and stored in dark at 4°C. XTT [2,3-*bis*(2-methoxy-4-nitro-5-sulfo-phenyl)-2H-tetrazolium-5-carboxanilide] was obtained as XTT sodium salt (Sigma Aldrich, MO, USA). Stock solution of XTT (1 mg mL^−1^ in PBS) was prepared and was used at a final concentration of 0.01% (w/v) in distilled water. The stock solution was filter sterilized in a 0.20* μ*m pore filter and stored at -20°C in dark.

### 2.3. Disk Diffusion Assay and Determination of MICs and MBCs

The antibacterial activities of ASHE and ASDE were assessed by disk diffusion method as previously described [25]. For disk diffusion assay, ASHE and ASDE were prepared freshly at a concentration of 10 mg mL^−1^ in 10% DMSO. Sterile antibiotic assay filter paper discs of 6 mm diameter were placed on MHA plates and 20* μ*L (10 mg mL^−1^ corresponding to 200* μ*g extract) of ASHE or ASDE was loaded onto the filter paper discs. Appropriate antibiotics were included as positive controls, while filter paper disc loaded with 20* μ*L of DMSO (10%) was included as a negative control (diluent control). The plates were incubated at 37°C and the inhibition zones were measured after 24 h incubation. The MICs and MBCs of ASHE and ASDE were determined by broth microdilution method and spread plate technique, respectively, by following the previously described methods [24, 26].

### 2.4. Time-Kill Assay

Time-kill assay was carried out on selected bacterial strains (MSSA, MRSA,* A. baumannii,* and* S. maltophilia*) according to the method described previously with minor modifications [26]. Bacterial suspensions were diluted to 1 × 10^6^ CFU mL^−1^. ASHE and ASDE concentrations were adjusted to 1x, 2x, and 4x MICs. Bacterial cultures treated with varying concentrations of the extracts were incubated at 37°C for 0, 0.5, 1, 2, 4, 8, 12, and 24 h. Aliquots of 100* μ*L were pipetted out from each tube at each time point, serially diluted in PBS, and spread-plated onto MHA plates. Tubes without ASHE and ASDE served as growth controls (0x). The plates were incubated at 37°C for 24 h followed by the enumeration of bacterial colonies. Killing curves were constructed by plotting the log_10_CFU mL^−1^ versus time over a 24 h time period. Bactericidal activity (99.9% kill) was defined as a ≥3-log_10_CFU mL^−1^ reduction in colony count from the initial inoculum.

### 2.5. Ultrastructure Microscopical Analysis

The ultrastructural changes in the bacteria treated with ASHE/ASDE were examined by SEM and TEM. Bacterial samples for electron microscopy were prepared by following the previously described method [27] with minor modifications. Cultures of* S. aureus*, MRSA,* A. baumannii*, and* S. maltophilia* were grown in TSB or BHI broth for 24 h at 37°C. The cultures were diluted to a final concentration of 5 × 10^7^ CFU mL^−1^ in broth (1:10) and aliquots of 5 mL were placed into each well of a 6-well polystyrene tissue culture plate. Samples were treated with varying concentrations of ASHE and ASDE (1x, 2x, and 4x MICs) for 4 h (based on the results obtained in time-kill studies) and cells were harvested by centrifugation and washed twice with 0.1 M PBS (pH 7.2) before proceeding for SEM and TEM analysis.

#### 2.5.1. Scanning Electron Microscopy (SEM)

Bacterial cells were fixed with buffered glutaraldehyde (4%) for 12-24 h, washed thrice with 0.1 M sodium cacodylate buffer, and postfixed in 0.1 M osmium tetroxide (OsO_4_) for 2 h at 4°C. Following fixation, samples were dehydrated in a graded acetone series (35-100%), mounted using double-sided tape, and subjected to critical point drying (CPD 030, Bal-TEC, Switzerland) and gold coating in a sputter coating unit (E5100 Polaron, UK). The specimens were examined in a SEM (JEOL JSM-6400, Japan) at 15 kV.

#### 2.5.2. Transmission Electron Microscopy (TEM)

Bacterial cells were fixed with glutaraldehyde and postfixed similar to the sample preparation as described for SEM in Section 2.5.1. Samples were dehydrated with a series of acetone grade (35%, 50%, 75%, 95%, and 100%) for 10-15 min each and infiltrated with increasing concentrations of acetone:resin mixture. Epoxy resin embedded samples were subjected to ultramicrotome and the ultrathin sections were double-stained with uranyl acetate and lead citrate. The specimens were examined under a Hitachi H-7100 TEM (Hitachi, Ibaragi) at 120 kV.

### 2.6. Antibiofilm Effect of ASHE and ASDE

#### 2.6.1. Antibiofilm Assay

Biofilms of* S. aureus*, MRSA,* A. baumannii*, and* S. maltophilia* were produced by microtitre plate method [28]. Briefly, overnight broth cultures of the bacterial samples were grown in BHI broth to a turbidity equivalent to 0.5 McFarland standard (10^8^ CFU mL^−1^). Each biofilm phenotype was added to 24 wells of a sterile microtitre plate and incubated at 37°C for 6 h under static conditions. After 6 h of adhesion, nonadherent cells were removed from each well and the wells were rinsed with 100* μ*L of physiological saline, and subsequently 100* μ*L of fresh medium was added to each well and incubated for 24 h. After 24 h of adhesion and biofilm formation, the supernatant was again removed and the wells were rinsed with physiological saline and 100* μ*L of ASHE and ASDE at concentrations of 1x, 2x, and 4x MICs were added. Wells without antibiotics or extracts were considered “untreated”. Untreated cells/negative controls were incubated with 100* μ*L of DMSO (5%) for an additional 12 h at 37°C with gentle shaking and the viability of biofilms was quantified by XTT-calorimetric assay.

#### 2.6.2. XTT Reduction Assay

The antibiofilm potential of ASHE and ASDE to inactivate biofilms was assessed by microtitre plate assay [29]. Before each assay, fresh XTT solutions were prepared by dissolving 4 mg of XTT (Sigma) in 10 mL prewarmed (37°C) PBS. This solution was supplemented with 100* μ*L menadione stock solution, containing 55 mg menadione (Sigma) in 100 mL acetone. The effect of ASHE and ASDE was tested at concentrations of 0x (untreated/negative control), 1x, 2x, and 4x MICs against 24 h old biofilms formed earlier. Posttreated biofilm plates were washed thrice with 200* μ*L of sterile PBS. The wells were dried and 100* μ*L of XTT-menadione solution was added to each well and incubated at 37°C in dark for 5 h. The contents of the wells were pipetted into fresh 1.5 mL Eppendorf tubes and centrifuged at 15,000* g* for 4 min. One hundred microlitres of the clear supernatant from each well was transferred to a sterile 96-well flat-bottomed microtitre plate and the absorbance of the adherent biofilm was read at 490 nm in a microplate reader (BioTek EL808, USA).

### 2.7. In Situ Visualization of the Antibiofilm Effects of ASHE and ASDE by Confocal Laser Scanning Microscopy (CLSM)

*In situ *antibiofilm potential of ASHE and ASDE on preformed biofilms of* S. aureus*, MRSA,* A. baumannii*, and* S. maltophilia* was visualized under a CLSM (Olympus FV1000-IX81). Biofilms were carefully washed with PBS and stained with LIVE/DEAD®BacLight Bacterial Viability Kit (Invitrogen, Grand Island, NY, USA). Equal volumes of live stain (green fluorescence) and dead stain (red fluorescence) were mixed and diluted to a working solution of 0.3% in sterile distilled water. The staining procedure was carried out according to the method described previously [30]. CLSM images were captured and processed using a Fluoview® FV1000 live cell imaging software.

### 2.8. Statistical Analysis

All assays were performed in triplicate (at a minimum) and repeated three times. Statistical analyses were performed using GraphPad Prism 6.0 software (GraphPad, San Diego, CA, USA) by one-way analysis of variance (ANOVA) with Dunnett's multiple comparison test. Values are expressed as ± SD. ^*∗∗∗*^*p* < 0.001 compared with the control.

## 3. Results

### 3.1. Antibacterial Activities of ASHE and ASDE

**Figure 1:**
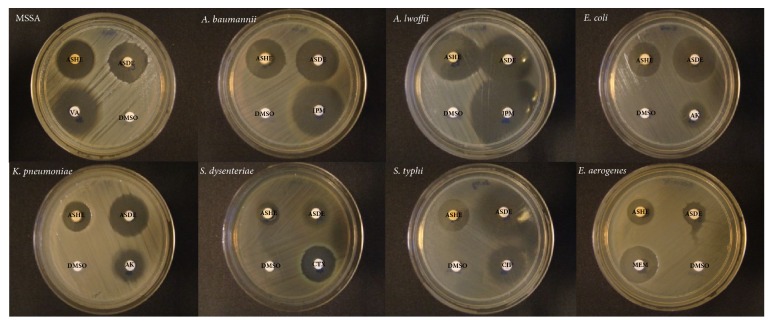
Effect of ASHE (200* μ*g disc^−1^) and ASDE (200* μ*g disc^−1^) applied to a blank filter paper disk on MHA plate inoculated with the test microorganisms. ASHE:* A. stipitatum* hexane extract; ASDE:* A. stipitatum* dichloromethane extract; VA: vancomycin (30* μ*g); IPM: imipenem (10* μ*g); AK: amikacin (30* μ*g); CTX: ceftriaxone (10* μ*g); CIP: ciprofloxacin (5* μ*g); MEM: meropenem (10* μ*g); DMSO: dimethyl sulfoxide (10%).

Both ASHE and ASDE showed promising antibacterial activity against all the bacteria tested and the inhibition zones ranged from 13 to 33 mm for ASHE and 15 to 32 mm for ASDE at 10 mg mL^−1^ concentration (Table 1). Both gram-positive and gram-negative organisms were susceptible to ASHE and ASDE. Of the 12 organisms tested, MDR pathogens, namely,* A. baumannii*, MRSA, and* S. maltophilia*, were highly susceptible to ASHE and ASDE with zone sizes ranging from 23 to 28 mm. The antibacterial results of MRSA were represented in this manuscript in order to facilitate better understanding for the readers. Meanwhile the diameter zone of inhibition for VRE and* P. aeruginosa* ranged from 13 to 15 mm (Figure 1). The largest zone of inhibition was observed in* A. lwoffii* exerted by ASHE (33 mm) at a concentration of 200* μ*g/disc. The MICs of ASHE and ASDE ranged from 16 to 1024* μ*g mL^−1^, while the MBCs ranged between 32 and 4096* μ*g mL^−1^ (Table 1). MSSA, MRSA, and* A. lwoffii* were found to be highly susceptible, especially at very low concentrations.

### 3.2. Analysis of Bacterial Killing Kinetics

**Figure 2:**
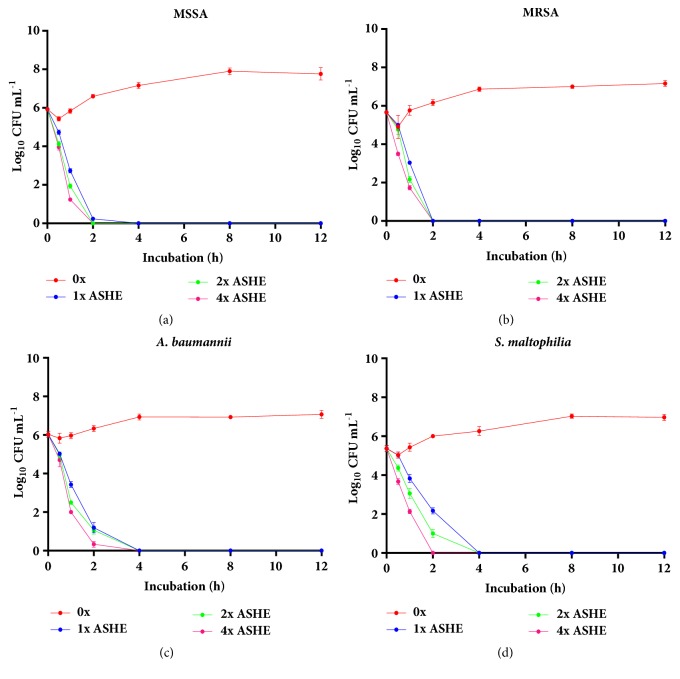
Effect of ASHE on the viability of (**a**) MSSA, (**b**) MRSA, (**c**)* A. baumannii*, and (**d**)* S. maltophilia* in liquid medium (time-kill curve) exposed with ASHE at concentrations of 1x, 2x, and 4x MIC with control (0x MIC).** MIC**: minimum inhibitory concentration;** CFU**: colony-forming units.

**Figure 3:**
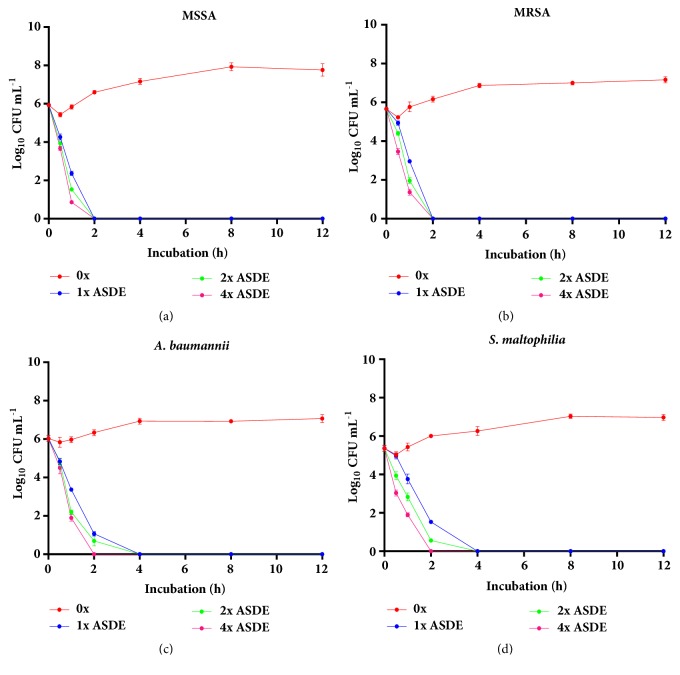
Effect of ASDE on the viability of (**a**) MSSA, (**b**) MRSA, (**c**)* A. baumannii*, and (**d**)* S. maltophilia* in liquid medium (time-kill curve) exposed with ASDE at concentrations of 1x, 2x, and 4x MIC with control (0x MIC).** MIC**: minimum inhibitory concentration;** CFU**: colony-forming units.

**Figure 4:**
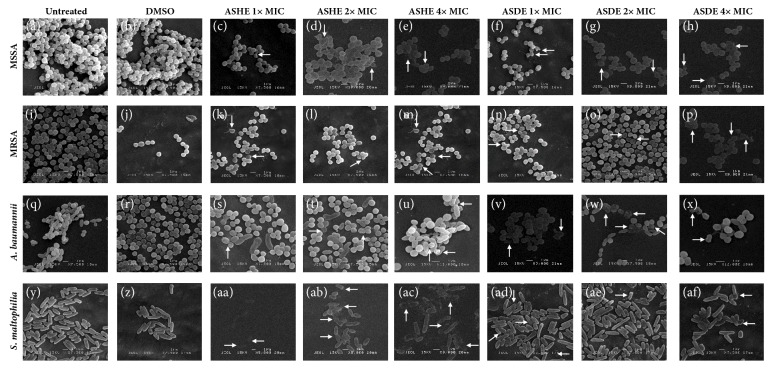
Scanning electron micrographs of test microorganisms treated with ASHE and ASDE. Panels (**a–h**) MSSA; (**i–p**) MRSA; (**q–x**)* A. baumannii*; (**y–af**)* S. maltophilia*. SEM magnification, ×5000 to ×12000. Bar indicates 1* μ*m.

Studies on the bacterial killing kinetics of ASHE and ASDE on MSSA, MRSA,* A. baumannii*, and* S. maltophilia* showed that the growth controls in MHB without any antibiotics (untreated) maintained their viability for 24 h. However, MSSA, MRSA,* A. baumannii*, and* S. maltophilia* treated with ASHE and ASDE at 1x, 2x, and 4x MICs showed a significant reduction in growth, indicating that both extracts were strongly bactericidal by killing >90% of the cells. Bactericidal endpoints were achieved at 2 h for MRSA (Figures 2(b) and 3(b)) and 4 h for MSSA (Figures 2(a) and 3(a)),* A. baumannii* (Figures 2(c) and 3(c)), and* S. maltophilia *(Figures 2(d) and 3(d)). At 1× and 4× MICs, the average reduction in CFUs was found to be ~1.9 and ~2.5 log units for ASHE and ASDE, respectively (99.9%).

### 3.3. ASHE and ASDE Treatments Caused Extracellular and Intracellular Damage to Test Bacterial Strains

#### 3.3.1. SEM

When treated with ASHE and ASHE at 1x, 2x, and 4x MICs for 4 h, several morphological changes in the cells were observed (Figure 4). Untreated and DMSO treated samples of MSSA (Figures 4(a) and 4(b)) and MRSA (Figures 4(i) and 4(j)) appeared in normal round shape with smooth cell surfaces.* Acinetobacter baumannii* (Figures 4(q) and 4(r)) cells appeared as smooth coccobacilli and* S. maltophilia* (Figures 4(y) and 4(z)) appeared normal rod shaped with clear smooth cell surfaces. Rough and damaged surfaces with bumps and ruptured lines on cell surfaces were observed in ASHE and ASDE treated samples of MSSA, MRSA, and* A. baumannii *(Figures 4(c)–4(f), 4(g), 4(k)–4(p), 4(t), and 4(u)). At higher concentrations of ASHE and ASDE (2x and 4x), burst cells of* S. maltophilia* were observed (Figures 4(ab) and 4(ac)). Lysed cells and cell debris of MSSA, MRSA,* A. baumannii*, and* S. maltophilia* were also observed (Figures 4(ab), 4(ac), 4(v)–4(x), and 4(ad)–4(af)).

#### 3.3.2. TEM

**Figure 5:**
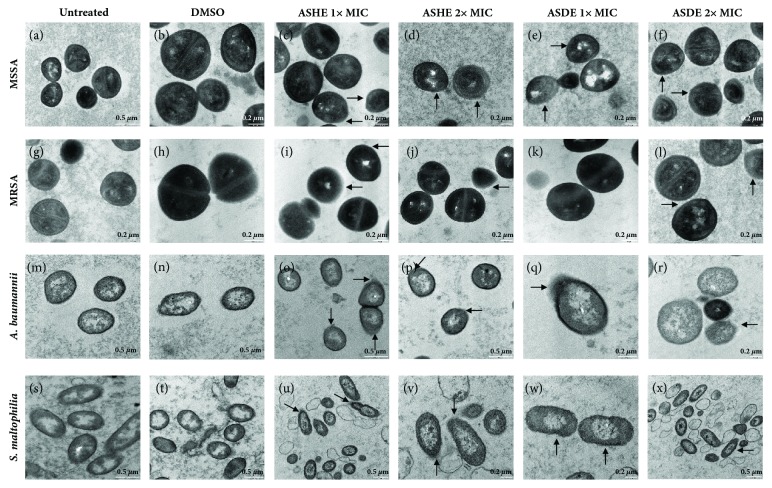
Transmission electron micrographs of test microorganisms treated with ASHE and ASDE. Panel (**a–f**) MSSA, (**g–l**) MRSA, (**m–r**)* A. baumannii*, and (**s–x**)* S. maltophilia*. SEM magnification, ×5000 to ×12000. Bar indicates 1* μ*m.

When treated with ASHE and ASHE at 1x and 2x MICs for 4 h, the intracellular damage and cell leakages were evident in extract treated samples (Figure 5). Nontreated and DMSO treated (control) samples of MSSA (Figures 5(a) and 5(b)) and MRSA (Figures 5(g) and 5(h)),* A. baumannii* (Figures 5(m) and 5(n)), and* S. maltophilia* (Figures 5(s) and 5(t)) showed intact cell shapes with homogenous cell wall and regular cell membranes. Disruption of cell membrane was observed in samples treated with 1x MIC (Figures 5(c)–5(f), 5(k), 5(l), 5(o)–5(q), 5(p), and 5(q)) and at increasing concentrations of ASHE/ASDE (2x MIC), ghost cells (Figures 5(i), 5(j), 5(l), 5(o), and 5(q)) and leakage of membrane components (Figures 5(o), 5(q), and 5(u)–5(x)) were observed.

### 3.4. The Effect of ASHE and ASDE on S. aureus, A. baumannii, and S. maltophilia Biofilms

**Figure 6:**
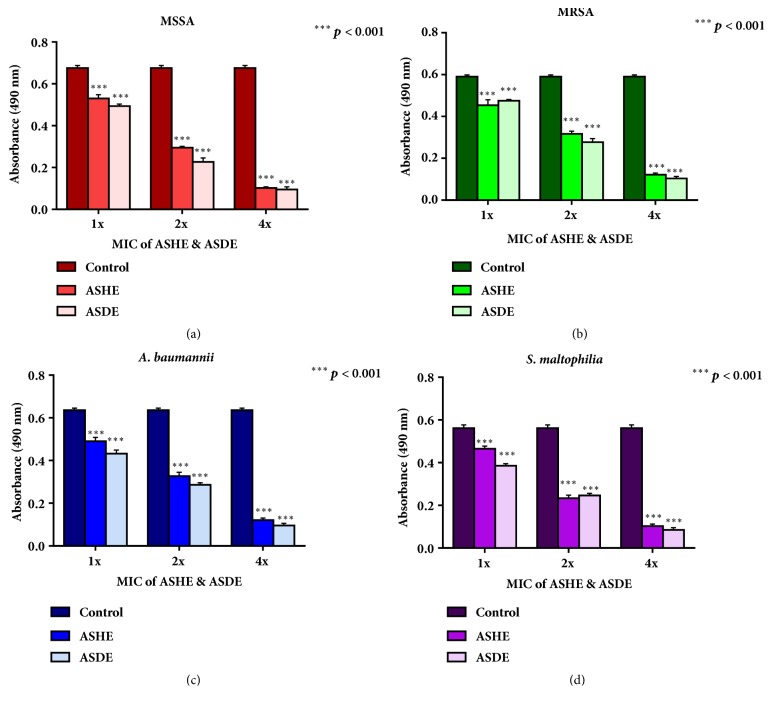
Effect of ASHE and ASDE on the viability of (**a**)* S. aureus*, (**b**) MRSA, (**c**)* A. baumannii*, and (**d**)* S. maltophilia* biofilms at concentrations of 1x, 2x, and 4x MICs with control (0x MIC). Comparison of absorbance between control and treated samples at 490 nm by XTT assay. ^*∗∗∗*^*p* < 0.001;** MIC**: minimal inhibitory concentration. Values are expressed as mean ± SD.

Challenging preformed biofilms of MSSA, MRSA,* A. baumannii*, and* S. maltophilia* with ASHE and ASDE resulted in significant reduction in biofilm viability. As shown in Figure 6, ASHE and ASDE disrupted/removed adherent biofilms at 1x, 2x, and 4x MICs in a concentration dependent manner. ASHE at 1× MIC showed slight reduction in the biofilm viability of MSSA (64* μ*g mL^−1^) (Figure 6(a)), MRSA (32* μ*g mL^−1^) (Figure 6(b)),* A. baumannii* (32* μ*g mL^−1^) (Figure 6(c)), and* S. maltophilia* (64* μ*g mL^−1^) (Figure 6(d)). Meanwhile, ASDE at 1× MIC showed a slight reduction in the biofilm viability of MSSA (16* μ*g mL^−1^), MRSA (64* μ*g mL^−1^),* A. baumannii* (32* μ*g mL^−1^), and* S. maltophilia* (64* μ*g mL^−1^). At 4x MICs, MSSA and* S. maltophilia* biofilms were highly susceptible to ASHE and ASDE (Figures 6(a) and 6(d)).

### 3.5. In Situ Analysis of Biofilm Formation

**Figure 7:**
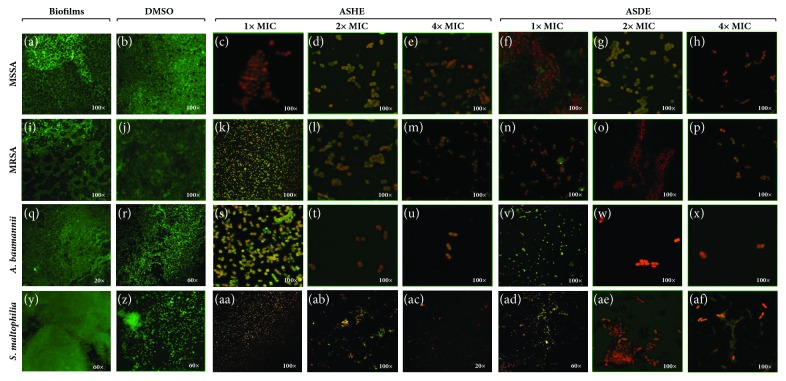
Representative confocal images of mature biofilms and ASHE and ASDE treated biofilms. Panels (**c–h**) represent ASHE and ASDE treated groups of MSSA. Panels (**k–p**) represent ASHE and ASDE treated groups of MRSA. Panels (**s–x**) represent ASHE and ASDE treated groups of* A. baumannii*. Panels (**aa–af)** represent ASHE and ASDE treated groups of* S. maltophilia*. Micrographs of fluorescent biofilms were obtained by confocal microscopy.

Figure 7 displays a series of CLSM images of the biofilms, before and after ASHE and ASDE treatments at varying concentrations. The controls (biofilms and DMSO treated) images on the 1^st^ and 2^nd^ columns as shown by the typical two-dimensional biofilm architectures of MSSA (Figures 7(a) and 7(b)), MRSA (Figures 7(i) and 7(j)),* A. baumannii* (Figures 7(q) and 7(r)), and* S. maltophilia* (Figures 7(y) and 7(z)) displayed well-developed biofilms (Figure 7), while bacterial samples treated with ASHE and ASDE at 1x MICs showed significant reduction in biofilms (Figures 7(a), 7(f), 7(k), 7(n), 7(s), 7(v), 7(aa), and 7(ad)). Meanwhile, CLSM images viewed at 20x, 60x, and 100x (in control biofilms and DMSO) unveiled the adhering ability of strong biofilm producers like MRSA,* A. baumannii*, and* S. maltophilia*, which led to the development of dense biofilm formation on glass coverslips. Meanwhile samples treated at increasing concentrations of ASHE and ASDE (2x and 4x MICs) clearly exhibited the antibiofilm potential of ASHE and ASDE by disintegrating the biofilm architecture of* S. aureus* (Figures 7(d), 7(e), 7(g), and 7(h)), MRSA (Figures 7(l), 7(m), 7(o), and 7(p)),* A. baumannii* (Figures 7(t), 7(u), 7(w), and 7(x)), and* S. maltophilia* (Figures 7(ab), 7(ac), 7(ae), and 7(af)).

## 4. Discussion

This is the first report on two different extracts from the same botanical product with antibacterial activity against MSSA, MRSA,* A. baumannii*, and* S. maltophilia* biofilms and their planktonic counterparts. The presence of allicin in the bulbs of* A. hirtifolium* (also known as Persian shallot locally) has been demonstrated by thin layer chromatography (TLC) methods [20]. In our earlier investigation on the* in vivo* antibacterial and burn wound healing properties of* A. stipitatum*, both ASHE and ASDE were subjected to gas chromatography mass spectrometer (GC-MS) analysis [24]. Although the antibacterial compound allicin was not detected in the GC-MS analysis, several sulfur-containing compounds including* S*-methyl methanethiosulfonate, 2,4,5-trithiahexane, 2,4-dithiapentane, 2-pyridinethione, and methane (chloromethylthio) (methylthio) were detected in our recently published work [24]. Similar components were also reported in the hydromethanolic extract of* A. hirtifolium* as detected by GMCS [21]. In their analysis, the hydromethanolic extract of* A. hirtifolium* was reported to be effective against MSSA, MRSA,* S. typhi*,* E. coli*, and* K. pneumoniae* with zone sizes ranging from 10 to 18 mm at 1.2 mg/disc concentration [21]. However, the use of nonpolar extracts (ASHE and ASDE) in this study resulted in a slightly higher activity. The strong antibacterial activity of ASHE and ASHE against the test pathogens can be attributed to the presence of volatile sulfur-containing compounds in the extracts as compared to the amount detected in the hydromethanolic extract reported elsewhere [21]. Another significance of the present study is the relatively equal potency exerted by ASHE and ASDE especially in killing gram-negative pathogens. Preliminary antibacterial data on the anti-MRSA activity of ASHE and ASDE based on disk diffusion and MIC experiments has been published recently by our research group [24]. Therefore, the disk diffusion plate picture for MRSA was not shown in Figure 1. However, the time-kill assay, electron microscopy images, and antibiofilm data of MRSA were included in the present study. Ismail et al. [21] reported that the inhibition zones exerted by hydromethanolic extract at 60 mg mL^−1^ towards gram-positive and gram-negative pathogens were in the ranges of 11-15 mm and 10-14 mm diameter, respectively. However, in the present study, ASHE at 10 mg mL^−1^ exerted zone sizes of 13-27 mm towards gram-positive bacteria and 13-33 mm towards gram-negative bacteria. Meanwhile, ASDE exerted zone sizes of 15-30 mm towards gram-positive bacteria and 15-32 mm towards gram-negative bacteria. Another significance of the present study is the use of emerging antibiotic resistant pathogens such as* A. baumannii*,* S. maltophilia*, and VRE which were highly susceptible to ASHE and ASDE. Based on the above data, it is evident that both ASHE and ASDE were equally potent in inhibiting both gram-positive and emerging gram-negative bacterial pathogens even at very low concentrations.

According to a previous study, the MIC values of hydromethanolic extract ranged from 1.88 to 7.50 mg mL^−1^ for gram-positive and gram-negative pathogens [21]. In the present study, ASHE and ASDE were strongly antibacterial and the MICs were as low as 32* μ*g mL^−1^ against epidemiologically important superbug like MRSA. The MICs of ASHE and ASDE were in the range of 16-1024* μ*g mL^−1^, while the MBCs ranged between 32 and 4096* μ*g mL^−1^. Such low levels of MICs for a natural product extract against emerging antibiotic resistant pathogens are noteworthy and advantageous compared with compounds of synthetic origin.

Few reports are available on the nontoxic antibacterial and cytoprotective effects of chloroformic and hydromethanolic extracts of* A. hirtifolium *[19, 21]. Moreover, in our earlier investigation on the anti-MRSA and burn wound healing properties of* A. stipitatum*, both extracts (ASHE and ASDE) were proven to be noncytotoxic and safe to Vero cells at <400* μ*g mL^−1^ [24]. In terms of antibacterial activity, the MICs of ASHE and ASDE against all gram-positive (except VRE) and gram-negative bacteria (except* Enterobacter* spp. and* P. aeruginosa*) were in the ranges of 16-256* μ*g mL^−1^ and 16-128* μ*g mL^−1^, respectively. The above MIC values were comparatively lesser than the CC_50_ concentrations on Vero cells (383.4* μ*g mL^−1^; 390.4* μ*g mL^−1^) which is therapeutically acceptable for a nontoxic edible natural extract. The cytotoxicity results as reported in our previous study [24] and the strong antibacterial activity of ASHE and ASDE at low concentrations clearly underline that nonpolar extracts of* A. stipitatum* can be successfully used as promising alternatives in managing gram-positive and gram-negative pathogens.

Several studies have reported on the preliminary antibacterial activity of* A. hirtifolium* [21, 31, 32]; however, investigation on the bactericidal effect of Persian shallot extract has never been reported elsewhere. Determination of the minimum time required by ASHE/ASDE in killing a pathogen will help in investigating the mechanism of action which involves studies like transcriptomic, gene expression analysis, and enzyme assays. Based on the data on time-kill assay, cell counts were found to be either too few to count (TFTC) or zero in MHA plates with bacterial inoculum plated after 4 h postinoculation. The colony counts of test bacteria treated with ASHE and ASDE at 2x and 4x MICs varied, but interestingly the killing time was the same for all the concentrations tested. The killing efficiency of both ASHE and ASDE towards* S. aureus*, MRSA,* A. baumannii*, and* S. maltophilia* was comparatively similar with slight variations in colony counts which could be due to the solvent's (dichloromethane) nature as a chlorinated solvent. No evidence of recurrence or growth was observed among the 4 pathogens tested which imply that the extracts are strongly bactericidal by completely killing the test pathogens in 2-4 hpi with an ~ 2 to 2.5 log reduction in the inoculum. The above findings are partly in agreement with Nidadavolu et al. [33] who reported ~ 7 log reduction in* A. baumannii*, ~ 8 log reduction in* S. aureus*, and ~ 2 log reduction in* Enterococcus faecalis *biofilms upon treatment with garlic oil. In addition, MDR strains of* A. baumannii* have been reported to be highly susceptible to chloroform extract of garlic [34]. Increasing concentrations of the extracts (2x and 4x MICs) did not show significant changes in the killing time, but the colony counts varied slightly which implies that the killing efficacy of ASHE and ASDE was concentration dependent.

Biofilm-related casualties due to* S. aureus*, MRSA,* A. baumannii*, and* S. maltophilia* are becoming more persistent in susceptible hospitalized patients with indwelling catheters or artificial grafts [35–37]. In the present study,* S. aureus* and* S. maltophilia* biofilms were more susceptible to ASHE and ASDE especially at 1x MIC levels (*p*< 0.001,* p*< 0.001) as compared to MRSA and* A. baumannii* (*p*< 0.001,* p*< 0.001), but statistically significant reduction in biofilm reduction was evident. One limitation of the present study is that the antibiofilm activities of ASHE and ASDE were tested only on mature biofilms (24-hour-old biofilm) and not on early biofilms (6-hour-old partial biofilm). In fact, mature biofilms are dense, thicker, and increasingly resistant to antibiotics as compared to early biofilms at 6 h. Both extracts were equally potential in penetrating deeper layers of the mature biofilms and effective in killing bacterial biofilms. Based on these results, it is evident that in addition to the broad-spectrum antibacterial activity towards planktonic bacteria, ASHE and ASDE possess antibiofilm activity on gram-positive and gram-negative biofilms. In an earlier investigation, Lee et al. [38] reported that garlic extract increases the biofilm formation by the oral biofilm colonizer* Streptococcus mutans* to orthodontic wire. However, the findings of the present study are contradictory, where* A. stipitatum *being a member of the* Allium* family disrupted matured biofilms of the MDR pathogens. According to Lee et al. [38], garlic oil (GarO) was shown to prevent biofilm development in burn wound pathogens such as* S. aureus* and* A. baumannii* [33]. These results were in agreement with the present study results implying that ASHE and ASDE could be possibly used as a prophylactic therapy to prevent biofilm associated infections upon further investigations.

Bacterial cell wall and cell membrane play a vital role in the survival, biofilm formation, and antibiotic resistance of a pathogen. Hence, a slight damage induced by a natural/synthetic antimicrobial compound could result in metabolic dysfunction/cell death. SEM and TEM experiments are highly essential techniques [27] in order to reveal any possible cell surface effects and/or intracellular alterations induced by ASHE/ASDE. It is evident from the SEM experiments that both gram-positive and gram-negative organisms tested showed signs of deep craters, burst, rough and damaged surfaces with depression, rupture lines, and cell debris. TEM experiments also showed clear disruption of bacterial membrane and leakage, which emphasize the bactericidal potentials of* A. stipitatum*.

Effective disruption and reduction of microcolonies were evidenced by CLSM images (Figure 7). The exact molecular mechanism underlying the growth and biofilm inhibition by ASHE and ASDE in MDR pathogens is not known; however, a recent investigation on the antibacterial effects of garlic (*Allium sativum*) concentrate and garlic-derived organosulfur compounds targets the cell membrane of* Campylobacter jejuni *[39]. Diallyl thiosulfinate (allicin) an active ingredient in garlic is known to inhibit RNA synthesis in* S. typhimurium* [40]. The use of crude plant extracts like ASHE/ASDE may be a valuable tool for future developments, also keeping in mind how economical it can be to obtain such preparations. However, further investigations must be carried out to define which of the many compounds from the multitarget crude mixtures is directed against which enzyme/protein.

## 5. Conclusion

*Allium stipitatum *is a source for bioactive compounds such as antioxidants, anticancer, and antimicrobial agents and has been shown here to have bioactive potential against pathogenic biofilms. The data presented here provides sufficient* in vitro* antibacterial activity and further extends the potential of* A. stipitatum *as an antibiofilm agent in addition to explaining the traditional use of Persian shallot as an antimicrobial agent. Hence, the combinatorial use of* A. stipitatum *extracts along with antibiotics could help to eradicate MDR pathogens and biofilm associated infections in hospital settings. Studies on the isolation of the bioactive compound and its mechanism of action are currently under investigation in our laboratory.

## Figures and Tables

**Table 1:** Zone of inhibition, MIC, and MBC of ASHE and ASDE against test microorganisms (20 *μ*L corresponding to 200 *μ*g/disc).

**Strain**	**Zone of inhibition in diameter (mm)** ^***a***^				**MIC (** ***μ*** **g mL** ^**-1**^ **)** ^***b***^		**MBC (** ***μ*** **g mL** ^**-1**^ **)** ^***c***^	
	**ASHE**	**ASDE**	**Antibiotic **	**DMSO (10**%**)**	**ASHE**	**ASDE**	**ASHE**	**ASDE**
Gram-positive bacteria								
MRSA^*∗*^	27 ± 0.5774	23 ± 0.2887	16 (VA)^*d*^	-	32	64	128	128
MSSA	26 ± 0.2887	30 ± 0.5774	30 (VA)	-	64	16	128	64
Vancomycin resistant enterococci	13 ± 0.7638	15 ± 0.8660	33 (TOB)^*e*^	-	512	1024	2056	4096
Gram-negative bacteria								
*Acinetobacter baumannii*	26 ± 0.2887	28 ± 0.0	33 (IPM)^*f*^	-	32	32	64	32
*A. lwoffii*	33 ± 0.5774	30 ± 0.2887	46 (IPM)	-	16	32	32	32
*Enterobacter *spp.	14 ± 0.2887	16 ± 0.5774	23 (MEM)^*g*^	-	512	1024	2056	4096
*Escherichia coli*	21 ± 0.2887	29 ± 0.5774	16 (AK)^*h*^	-	128	32	512	128
*Klebsiella pneumoniae*	20 ± 0.0	29 ± 0.5000	- (AK)	-	256	64	512	256
*Pseudomonas aeruginosa*	13 ±0.5774	15 ± 0.2887	23 (CAZ)^*i*^	-	1024	1024	2056	4096
*Salmonella typhi*	20 ± 0.5000	32 ± 0.5000	25 (CIP)^*j*^	-	256	32	256	128
*Shigella dysenteriae*	18 ± 0.7638	22 ± 0.5000	27 (CTX)	-	256	128	1024	1024
*Stenotrophomonas maltophilia*	26 ± 0.5774	27 ± 1.000	30 (SXT)^*k*^	-	64	64	256	256

^*a*^Determined by disk diffusion assay.

^*b*^Determined by broth microdilution method.

^*c*^Determined by plate colony count technique.

^*d*^Vancomycin (30 *μ*g).

^*e*^Tobramycin (10 *μ*g).

^*f*^Imipenem (10 *μ*g).

^*g*^Meropenem (30 *μ*g).

^*h*^Amikacin (30 *μ*g).

^*i*^Ceftriaxone (10 *μ*g).

^*j*^Ciprofloxacin (5 *μ*g).

^*k*^Trimethoprim-sulfamethoxazole (30 *μ*g).

-: no zone of inhibition.

^*∗*^Preliminary antibacterial data for ASHE/ASDE on MRSA (disk diffusion and MIC values) has been published recently by our research group (Karunanidhi et al., 2017).

## Data Availability

The data used to support the findings of this study are available from the corresponding author upon request.

## Abbreviations

ASHE: * Allium stipitatum* hexane extract
ASDE: * Allium stipitatum* dichloromethane extract
MSSA: Methicillin-sensitive* Staphylococcus aureus*
MRSA: Methicillin-resistant* Staphylococcus aureus*
MIC: Minimum inhibitory concentration
MBC: Minimum bactericidal concentration
XTT: 2,3-*Bis*(2-methoxy-4-nitro-5-sulfophenyl)-*2H*-tetrazolium-5-carboxanilide
CLSM: Confocal laser scanning microscopy
MDR: Multidrug resistant
hpi: Hours postinoculation
ATCC: American Type Culture Collection
TSA: Tryptic soy agar
TSB: Tryptic soy broth
NA: Nutrient agar
LB: Luria-Bertani
BHI: Brain heart infusion
MHA: Mueller-Hinton agar
MHB: Mueller-Hinton broth
PBS: Phosphate-buffered saline
CLSI: Clinical and Laboratory Standards Institute
CFU: Colony-forming units
SEM: Scanning electron microscopy
TEM: Transmission electron microscopy
CPD: Critical point drying
TLC: Thin layer chromatography
GC-MS: Gas chromatography mass spectrometer
VRE: Vancomycin resistance enterococci
TFTC: Too few to count
RNA: Ribonucleic acid.
